# Effects of Dietary Supplementation with Fermented *Zanthoxylum schinifolium* Leaves on Growth Performance, Meat Quality, and Sensory Traits in Sanhuang Chicken

**DOI:** 10.3390/foods14142542

**Published:** 2025-07-21

**Authors:** Yi Zhang, Mingze Fu, Gang Yang, Xiaowei Peng, Hongwei Wang, Jianquan Kan

**Affiliations:** 1 College of Food Science, Southwest University, Chongqing 400715, Chinafmz000623@163.com (M.F.);; 2 Chongqing Key Laboratory of Speciality Food Co-Built by Sichuan and Chongqing, Chongqing 400715, China; 3 Chinese-Hungarian Cooperative Research Centre for Food Science, Chongqing 400715, China

**Keywords:** *Zanthoxylum schinifolium* leaves, fermented feed, growth characteristics, quality, sensory

## Abstract

Incorporating specific nutritional supplements into animal diets can significantly enhance the quality and various characteristics of animal meat. This study investigated the effects of fermented *Zanthoxylum schinifolium* leaves (ZSLs) on growth performance, meat quality, and sensory attributes in Sanhuang chickens. Three hundred one-day-old Sanhuang chickens were randomly divided into five groups and reared for 70 days: NC (control, basal diet), NF (6% unfermented ZSLs), LDG (3% fermented ZSLs), MDG (6% fermented ZSLs), and HDG (9% fermented ZSLs). Supplementation with 6% fermented ZSLs significantly increased the leg muscle percentage by 7.4% and decreased the abdominal fat percentage by 22.6%. Meat quality improved notably in MDG, with higher levels of polyunsaturated fatty acids, particularly n-3 polyunsaturated fatty acids. Increasing the proportion of fermented ZSLs enhanced the levels of umami amino acids and sweet amino acids by 36.5% and 11.6%, respectively. Additionally, the enhancement of aroma and flavor of chicken may be correlated with supplementation of fermented ZSLs. These results establish fermented ZSLs as a valuable feed additive for improving production efficiency and meat quality in Sanhuang chickens.

## 1. Introduction

Soybean meal, valued for its high yield and rich amino acid content, is the primary protein source in the feed industry [1]. However, population growth and decreasing per capita arable land have disrupted the supply–demand balance, intensifying protein shortages and increasing soybean meal prices [2]. These rising costs have created significant challenges for Asia’s livestock sector, highlighting the critical need for cost-effective and sustainable protein alternatives to ensure long-term industry stability.

Herbal plants are increasingly used as feed additives for their antibacterial, antiparasitic, antioxidative, immune-modulatory, and growth-promoting properties [3]. Among these, *Z. schinifolium*, a woody plant of the Rutaceae family, stands out for its edible and medicinal applications. Its leaves (ZSLs) are rich in bioactive compounds, including polyphenols and flavonoids, which confer antibacterial, antioxidant, and antiparasitic effects [4]. Despite these benefits, ZSLs are predominantly discarded or burned, with minimal use as fertilizer, leading to low utilization rates and environmental pollution. ZSLs contain approximately 20% protein. ZSLs are a high-quality and readily accessible feed resource with significant development potential. However, their high crude fiber content (15.83%) limits direct use in poultry feed due to associated health risks [5]. Poultry exhibit a considerably limited ability to digest crude fiber due to the absence of endogenous hydrolytic enzymes. Furthermore, soluble non-starch polysaccharides (NSPs) substantially elevate digesta viscosity, thereby impairing nutrient digestibility in the small intestine [6] (Li et al., 2023).

Solid-state fermentation is an effective technique for enhancing the quality of unconventional feed at present [7]. Through this process, microorganisms produce enzymes that degrade crude fiber and anti-nutritional factors, enriching the feed with probiotics, organic acids, and functional enzymes. For instance, Shuai et al. [8] showed that fermented rapeseed meal improves the growth performance and antioxidant capacity in pigs, while Kokoszyński et al. [9] observed similar benefits from fermented corn silage on goose carcass quality and production. Moreover, dietary modifications can enhance meat quality and influence flavor-related metabolites [10]. Hu et al. [11] discovered that the addition of ZBL powder in the diet improved the growth performance and antioxidant capacity of broilers via the Nrf2/HO-1 signaling pathway. Currently, the application of ZSLs in animal husbandry is steadily increasing, although it remains largely confined to directly feeding meat rabbits and laying hens with ground leaf powder. However, the application of fermented ZSLs in animal production has scarcely been explored.

Sanhuang chicken, a yellow-feathered broiler named for its yellow feathers, beak, and feet, is widely raised in southern China [12]. Known for its distinctive meat flavor and color, this breed is prized for its high meat yield, stress resilience, and reproductive performance [13]. Due to its slower growth rate, the meat has a firmer texture and a richer flavor, making it ideal for a variety of cooking methods. It plays a pivotal role in Chinese poultry production and has also influenced European breeding programs [14]. Despite its significance, the effects of fermented ZSLs on Sanhuang chickens remain understudied. We therefore aim to substitute a portion of the basal diet’s protein component with fermented ZSLs, reducing feed costs while enhancing poultry growth performance through improved nutrient bioavailability. This study evaluated the effects of different inclusion levels of fermented ZSLs on growth performance, carcass traits, meat quality, and sensory attributes in Sanhuang chickens. This study provides the first comprehensive investigation into the effects of dietary fermented ZSL supplementation on meat quality traits in Sanhuang chicken, delivering key mechanistic insights for optimizing ZSL applications in poultry production to enhance meat quality.

## 2. Materials and Methods

### 2.1. Feed Preparation and Component Analysis

Basic commercial chicken feed was purchased from Chongqing Sanwang Feed Co., Ltd. (Chongqing, China), with its detailed ingredients and nutritional composition provided in Table S1. ZSLs, a byproduct of Sichuan pepper production, consist of mature leaves harvested in late May and were procured from Chongqing Jiaofeng Agricultural Development Co., Ltd. (Chongqing, China). A fermentation agent specifically formulated for straw and feed fermentation was supplied by Zhengzhou Sannong Chutian Biological Technology Co., Ltd. (Henan, China). This agent mainly consisted of probiotics, including lactic acid bacteria, yeast, and *Bacillus* spores. Our findings, currently unpublished, showed that different fermentation agents and durations significantly affect the fermentation quality of ZSLs. Using the membership function method to assess various quality indicators, we found that the highest membership value was achieved after 15 days of fermentation with this agent, indicating the optimal fermentation outcome. Additionally, we optimized the fermentation process, with the final conditions being a 0.6% agent addition, 60% moisture content, and a fermentation temperature of 33 °C. The ZSLs were fermented with the commercial fermentation agent in accordance with the optimized fermentation process, and the basal diet was thoroughly mixed with either ZSLs or fermented ZSLs in the proportions defined by the experimental design before being fed to the chickens. The mixture was prepared using 9% fermented ZSLs as an example, consisting of 91% basal diet and 9% fermented ZSLs (*w*/*w*). The component analysis results of ZSLs and fermented ZSLs are shown in Table 1.

A non-targeted metabolomics analysis of plant bioactive compounds was carried out using liquid chromatography–mass spectrometry (LC-MS/MS) on a Vanquish UHPLC system (Thermo Fisher Scientific, Waltham, MA, USA) equipped with a Hypesil Gold column (1.9 μm, 100 × 2.1 mm). This analysis aimed to identify bioactive compounds formed during the ZSL fermentation process. Testing services were provided by Genedenovo Biotechnology Co., Ltd. (Guangzhou, China).

### 2.2. Animal Feeding and Sample Collection

The animal care and use protocol for this study was approved by the Institutional Animal Care and Use Committee of Southwest University (Chongqing, China; approval no. IACUC-20240410-01). The institutional ethical guidelines have been placed in the Supplementary Materials. The Sanhuang chickens used in this experiment were procured from Chongqing Changshui Poultry Industry Development Co., Ltd. (Chongqing, China). The chickens were raised following the ‘Feeding standard of chicken’ (NY/T 33-2004) issued by the Ministry of Agriculture and Rural Affairs of the People’s Republic of China. Three hundred one-day-old Sanhuang chickens were reared under free foraging conditions for 10 days. Chickens of similar size and weight were then randomly assigned to five groups, each consisting of 60 chickens further divided into six blocks of 10 chickens, half of which were male and the other half female. The groups were categorized by diet as NC (basal diet), NF (6% unfermented ZSLs), LDG (3% fermented ZSLs), MDG (6% fermented ZSLs), and HDG (9% fermented ZSLs). The chickens were raised under these dietary regimens until 71 days of age. Feeding was conducted twice daily at 9 a.m. and 5 p.m., with ad libitum access to both feed and water throughout the study. The experiment spanned 70 days, including a 10-day adaptation period followed by a 60-day feeding trial. Average daily gain (ADG) and average daily feed intake (ADFI) were calculated using average body weight (ABW) measurements recorded on days 1 and 71 and total feed consumption per group. The feed conversion ratio was calculated as the ratio of ADFI to ADG (F: G).

### 2.3. Carcass Traits

At the end of the feeding trial (71 days), six chickens (three males and three females) were randomly selected from each group for live weighing measurement and subsequent slaughter. The selected chickens were slaughtered via the prescribed procedures: the Sanhuang chickens were stunned using electric shock and subsequently bled to ensure humane slaughter. Immersion in hot water at 65–75 °C for 1–2 min was performed to facilitate defeathering, after which the dressing weight was recorded. The semi-eviscerated weight was measured after removing the trachea, esophagus, crop, intestines, spleen (without the gallbladder), pancreas, and reproductive organs, while retaining the heart, liver, lungs, kidneys, proventriculus, gizzard, and abdominal fat. The eviscerated weight was determined by further removing the heart, liver, proventriculus, gizzard, abdominal fat, head, neck, and feet, with the lungs and kidneys remaining intact. The breast muscle, leg muscle, and abdominal fat weights were recorded. Subcutaneous fat thickness (SFT) was measured using a vernier caliper. Dressing percentage (DP), semi-eviscerated percentage (SEP), and eviscerated percentage (EP) were calculated as the ratio of their respective weights to live weight. Breast muscle percentage (BMP), leg muscle percentage (LMP), and abdominal fat percentage (AFP) were calculated as the ratio of their respective weights to EP.

### 2.4. Meat Quality Assessment

#### 2.4.1. Chemical Composition and Nutritional Value

Intramuscular fat (IMF) content analysis: The IMF content of chicken breast muscle was analyzed according to Chinese standard GB 5009.6–2016 for fat determination in food. A 5 g sample was placed in a filter paper cartridge and subjected to reflux extraction in a Soxhlet extractor using petroleum ether for 6 h. The solvent was subsequently recovered, evaporated to dryness, and the remaining residue was weighed after cooling.

MP content was measured using the Kjeldahl method. A 2 g pulverized sample of chicken breast muscle was transferred to a digestion tube. Copper sulfate (0.4 g), potassium sulfate (6 g), and sulfuric acid (20 mL) were added, and the mixture was digested in a digestion furnace. Following digestion, titration was completed using an automatic Kjeldahl nitrogen analyzer (K1100F, Shandong Haineng Scientific Instrument Co., Ltd., Shandong, China).

Fatty acid composition analysis: The fatty acid content was analyzed following the Chinese standard GB 5009.168–2016 (determination of fatty acids in food). Fat was extracted from 3 g of freeze-dried chicken tissue using Soxhlet extraction. The extracted fat was treated with 7 mL of acidified methanol and subjected to condensation reflux to complete methylation. The methylated product was then extracted with 10 mL of 1-heptane, and saturated aqueous sodium chloride solution was added to facilitate phase separation. A 5 mL aliquot of the upper layer was combined with 3 g of anhydrous sodium sulfate to remove residual moisture. Finally, the upper solution was collected and analyzed using a gas chromatography (7890A, Agilent Technologies, Santa Clara, CA, USA) system equipped with an SP-2560 capillary column (100 m × 0.25 mm × 0.2 µm, Supelco, Bellefonte, PA, USA) and a flame ionization detector. The GC injector temperature was 270 °C, and the detector temperature was 280 °C. The column chamber was initially maintained at 100 °C for 13 min, and the temperature was then increased to 180 °C at a rate of 10 °C/min and maintained for 6 min. The temperature was then increased to 200 °C at a rate of 1 °C/min for 20 min, finally increased to 230 °C at a rate of 4 °C/min, and maintained for 10.5 min. Nitrogen was used as the carrier gas, and 1 µL of sample was injected, with a split ratio of 100:1. Fatty acids were identified by comparing retention times with the 37 component fatty acid methyl esters standards and quantified using methyl salicylate (500 ppm) as the internal standard.

Free amino acid analysis: The free amino acid (FAA) content was measured using 1 g of lyophilized sample dissolved in water and diluted to 50 mL. A 4 mL aliquot of the solution was mixed with 4 mL of sulfosalicylic acid to precipitate proteins. The resulting filtrate was treated with 2 mL of EDTA-2Na (1%, *w*/*w*) and 2 mL of hydrochloric acid solution (0.06 mol/L) and allowed to react for 20 min. The mixture was centrifuged at 12,000× *g* for 15 min at 4 °C to collect the supernatant. The supernatant was filtered through a 0.22 µm Millipore nylon filter and analyzed using an automatic amino acid analyzer (L-8900, Hitachi, Tokyo, Japan).

#### 2.4.2. Physicochemical Composition

The pH of chicken breast muscle was determined at 45 min and 24 h postmortem. A tip electrode from a PHS-3C bench-top pH meter (Leici, INESA Co., Ltd.; Shanghai, China) was inserted 2 cm into the midpoint of the muscle. Stable readings were recorded, with six measurements conducted for each group.

Color measurement: The color of chicken breast muscle was evaluated 24 h postmortem using a CM-5 chromameter (Minolta Camera, Osaka, Japan). Lightness (L*), redness (a*), and yellowness (b*) were measured at the upper, middle, and lower sections of the same breast muscle side. Before measurement, the device was calibrated with a white calibration plate. Each group was measured independently six times.

Drip loss measurement: Determined with reference to Zaremba et al. (2024) [15], uniformly sized chicken breast samples were selected and suspended in plastic cups using wires, with one end of each wire attached to the sample and the other secured to the bottom of the cup. After 24 h, surface moisture was removed with paper towels. The drip loss rate was calculated using the following formula: (Weight of the samples before suspension-Weight of the samples after suspension)/Weight of the samples before suspension ×100%.

Cooking loss measurement: Rectangular meat samples measuring 3 × 3 × 1 cm and weighing approximately 15 ± 1 g were prepared by removing connective tissue. The samples were sealed in high-temperature cooking bags and cooked in a water bath at 75 °C for 15 min. After cooking, the samples were cooled to room temperature, and surface moisture was removed with paper towels. The cooking loss rate was calculated as follows: (Weight of the sample before cooking-Weight of the sample after cooking)/Weight of the samples before cooking ×100%.

Shear force analysis: Three subsamples were extracted from each cooked sample, cut parallel to the muscle fibers with a cross-section of 1 × 1 cm. Shear force was measured perpendicular to the muscle fibers using a texture analyzer (TA-XT plus, SMS Co., Ltd.; London, UK) equipped with an HDP/BS dovetail blade. The parameters were set as follows: pre-test, test, and post-test speeds of 5 mm/s, 1 mm/s, and 5 mm/s, respectively; a trigger force of 5 g; and a penetration depth of 10 mm. Each subsample was tested three times, and the results are expressed as the mean of the three measurements.

#### 2.4.3. Sensory Assessment

This research was approved by the Ethics Committee of Food Science, Southwest University (Chongqing, China; approval no. HF20240407). The privacy of the subjects was respected during the experiment, and informed consent was obtained from them. The texture, flavor, and taste of chicken were assessed using descriptive sensory analysis (DA). A panel of 10 trained assessors (5 men and 5 women, aged 20–25) conducted the evaluations after completing 10 training sessions over four weeks to familiarize themselves with the sensory attributes of boiled chicken breast. The evaluated attributes included juiciness, cohesiveness, springiness, chewiness, hardness, fibrousness, denseness, fat odor, smoked odor, sulfurous odor, and nut-like, sour, cardboardy, salty, umami, astringent, and metallic taste. Reference control samples are listed in Table S2. The assessment followed a modified version of the method described by Xu et al. [16]. Chicken breasts were cut into uniformly sized pieces, placed in sealed plastic cups, and maintained at 60 °C on a heating plate to replicate typical dining temperatures. Each sample was coded with a randomized three-digit number and presented to panelists. Texture and taste were evaluated after chewing each sample 5–10 times. Flavor was assessed by first sniffing reference samples to establish baseline aromas, followed by closed-eye sniffing of the test samples. Panelists were instructed to avoid communication, abstain from wearing scented products or makeup, and rinse their mouths between samples to prevent cross-contamination and residual interference.

#### 2.4.4. Electronic Tongue Analysis

Chicken samples (15 g) were chopped and diluted with ultrapure water at a 1:10 material-to-liquid ratio. The mixture was sequentially filtered through qualitative filter paper and a 0.45 μm microporous membrane to produce a clear filtrate. The prepared filtrate was analyzed using an electronic tongue system (TS-SA402B, INSENT, Atsugi, Japan).

#### 2.4.5. Electronic Nose Analysis

Chicken breast samples (8 g) were placed in 50 mL centrifuge tubes and equilibrated for 30 min prior to testing. A headspace sampling approach was employed using the electronic nose (PEN 3.5, Airsense Inc.; Shiweilin, Germany), which features 10 highly sensitive heated metal oxide detectors. The detection conditions were configured as follows: a sampling time of 1 s per group, a cleaning duration of 100 s, an injection time of 5 s, and an injection flow rate of 400 mL/min.

#### 2.4.6. Volatile Compound Identification Based on GC–MS Analysis

Boiled chicken breast samples (1 g) were placed in 20 mL headspace vials. To each vial, 10 µL of 1.6 mg/mL 2-octanol, serving as the internal standard, was added before sealing. The vials were equilibrated at 60 °C for 20 min. Subsequently, a solid-phase microextraction (SPME) needle was inserted, and the fiber tip was exposed to the headspace for 30 min to adsorb volatile compounds. The fiber tip was then transferred to the injection port of a GC-MS (Agilent 8890-5977B, Thermo Fisher Scientific, Waltham, MA, USA) for a 5 min analysis. Chromatographic conditions were optimized as follows: An HP-5MS column (30 m × 0.25 mm × 0.25 µm) was employed, with helium as the carrier gas at a flow rate of 1 mL/min under splitless injection. The column temperature was initially set at 40 °C and held for 3 min, increased by 3 °C/min to 70 °C, then by 5 °C/min to 180 °C, and finally by 10 °C/min to 280 °C, where it was maintained for 5 min. For qualitative analysis, MS results were compared against a compound library, with a minimum match score of 80 (maximum score: 100). External retention index calibration was performed using a mixture of n-alkanes (C7–C40). Quantitative analysis was carried out using the internal standard method, with the relative concentrations of volatile compounds calculated from the known concentration of 2-octanol and the corresponding peak areas.

### 2.5. Statistical Analysis

All experiments were conducted in triplicate, and the results are expressed as mean ± standard deviation (SD). Statistical analysis was performed using one-way Analysis of Variance (ANOVA) in SPSS 27 software (IBM, Armonk, NY, USA), followed by Duncan’s multiple range test. Prior to ANOVA, data were checked for normal distribution and homogeneity of variance. Graphs were created using Origin 2021 (Origin Co., Ltd., Farmington, ME, USA).

## 3. Results and Discussion

### 3.1. Metabolite Profile of Fermented ZSLs

Analysis of differential metabolites before and after fermentation (Figure S1) showed a notable increase in flavonoids such as vitexin, catechin, kaempferol, and poncirin, which are key contributors to antioxidant activity. These results align with Xu et al. [17], who reported that flavonoids and flavanones in sainfoin silage serve as critical differential metabolites. This increase likely stems from microbial enzyme activity during fermentation, which facilitates the release of active compounds from plant cell walls.

Oxidative stress negatively impacts animal productivity and meat quality [18]. Polyphenol-rich plant supplements and fermented feeds provide natural protection against oxidative stress, thereby improving performance and meat quality [19]. For example, *B. papyrifera* silage enhances antioxidant status due to its bioactive components, including flavonoids, phenolic acids, and alkaloids [20]. Similarly, our study found that fermented ZSLs are rich in bioactive compounds and protein, making them a viable and sustainable protein source for livestock, including Sanhuang chickens. Dietary inclusion of fermented ZSLs has the potential to improve growth performance and meat quality in Sanhuang chickens. This study highlights the value of utilizing *Z. schinifolium* by-products, offering a sustainable strategy for enhancing animal nutrition and feed production.

### 3.2. Growth Performance and Carcass Characteristics

As shown in Table 2, initial body weight (ABW1) did not differ significantly between the control and treatment groups (*p* > 0.05). However, dietary supplementation with fermented ZSLs significantly enhanced feed intake, final body weight (ABW2), ADG, and ADFI, while reducing the feed-to-gain ratio (F: G) (*p* < 0.05), thereby improving feed efficiency. The MDG group showed the highest ADG, followed by HDG, with feed efficiency significantly higher in MDG than in the control group (NC) (*p* < 0.05). Carcass traits, DP, was higher in the treatment groups than in NC. Additionally, AFP was significantly lower in MDG compared to NC (*p* < 0.05). These results suggest that supplementation with 6% fermented ZSLs improved growth performance and carcass traits, simultaneously reducing the abdominal fat percentage and avoiding excessive abdominal fat deposition in Sanhuang chickens, thereby improving feed utilization efficiency. Studies have demonstrated that excessive fermented feed generates high concentrations of lactic acid and acetic acid, which disrupt intestinal acid–base balance and depress digestive enzyme activity. Furthermore, the accumulation of certain anti-nutritional factors directly compromises the intestinal mucosal barrier, thereby inhibiting poultry growth.

Similar studies have reported that incorporating 5%, 10%, or 15% fermented feed based on cottonseed and rapeseed meal into broiler diets significantly enhances growth performance [21]. The improved performance of Sanhuang chickens in this study may be attributed to the superior protein quality and biological value of ZSLs, which aligns closely with animal protein profiles. Fermentation further enhances nutrient digestibility, particularly amino acids [22]. Our previous study demonstrated that fermentation increased ZSL digestibility by raising the cellulose degradation rate to 42.86% and boosting acid-soluble protein content by 5.14-fold, facilitating nutrient absorption. Fermentative microorganisms secrete a spectrum of digestive enzymes, including proteases and cellulases. Acid proteases exhibit elevated metabolic activity in acidic environments, facilitating protein degradation that increases acid-soluble protein (ASP) content, thereby enhancing feed utilization efficiency. Consistent with Ashayerizadeh et al. [23], fermented feed may also enhance gut health similarly to probiotics. The improved dressing percentage and carcass traits in treatment groups likely result from increased intake of bioavailable nutrients, supporting superior growth performance. These results align with previous findings showing that fermented feed supplementation enhances broiler growth rates [24].

### 3.3. Meat Quality Parameters

Table 3 indicates that intramuscular fat content was significantly higher in the MDG and HDG groups compared to NC (*p* < 0.05). Muscle protein content and pH45min values showed no significant differences between the treatment and control groups (*p* > 0.05). After 24 h, MDG and HDG displayed higher pH values and lower a* and b* values than the control group (*p* < 0.05). The HDG group had the lowest cooking and drip loss rates, followed by MDG (*p* < 0.05). Shear force was significantly greater in NC compared to all treatment groups (*p* < 0.05).

Meat quality is a multifaceted evaluation of characteristics that shape consumer preferences [25]. Higher intramuscular fat content enhances muscle juiciness, flavor, and tenderness while it lowers shear force [26]. This study corroborates these findings, as the treatment groups showed increased intramuscular fat and decreased shear force. Similarly, Xu et al. [27] demonstrated that supplementing pork with 800 mg/kg of apple polyphenols reduced L* and b* values. In this study, ZSL supplementation significantly decreased the a* and b* values in chicken meat. This reduction may result from the oxidation of myoglobin, which transforms bright red oxymyoglobin into brown metmyoglobin, affecting redness [28]. Additionally, polyphenols can bind to proteins, reducing their chromogenic potential [29]. Quinones generated from polyphenol oxidation can form covalent bonds with myosin sulfhydryl groups (-SH), thereby masking the chromophoric heme iron site of myoglobin. Additionally, polyphenols may bind iron atoms to accelerate metmyoglobin accumulation, adversely impacting meat redness. ZSLs contain functional plant secondary metabolites, such as flavonoids, which inhibit free radical formation. The bioactive compounds in ZSLs likely contributed to the observed changes in meat color. Natural antioxidants, like those in ZSLs, stabilize cell membranes and enhance the water-holding capacity of meat [30]. Thus, incorporating ZSLs may improve water retention and tenderness in chicken.

### 3.4. Fatty Acid Composition

No significant differences were observed in C18:1n9t and C20:3n6 levels among the groups (*p* > 0.05; Table 4). However, C14:1 and C20:2 levels were significantly higher in all treatment groups compared to the control group (*p* < 0.05). Fermented ZSL supplementation significantly increased C18:0 and C22:6n3 levels in the treatment groups relative to NC and NF (*p* < 0.05), while C16:0, C16:1, C18:2n6c, and C20:4n6 levels were significantly lower than in the control (*p* < 0.05). Total saturated fatty acids (SFAs) were highest in NC (*p* < 0.05). In contrast, MDG had significantly higher levels of C18:0, C20:2, and polyunsaturated fatty acids (PUFAs) compared to the other treatment groups (*p* < 0.05). NC exhibited the highest total n-6 PUFA content and the lowest n-3 PUFA content (*p* < 0.05), whereas MDG had the highest total n-3 PUFA levels (*p* < 0.05).

Previous studies have demonstrated that plant-based diets rich in natural antioxidants can increase n-3 PUFA concentrations in meat [31]. Marcinčák et al. [32] reported that supplementing poultry feed with 10% fermented corn meal significantly elevated the proportions of oleic acid, α-linolenic acid, and γ-linolenic acid in chicken breast fat while concurrently improving the n-3/n-6 PUFA ratio. Kwiecień et al. [33] revealed that thigh muscle exhibited the highest n-3 PUFA content when supplemented with low-dose alfalfa (*Medicago sativa*), which demonstrated antioxidant potential capable of modulating fatty acid profiles. In this study, supplementation with 6% fermented ZSLs enhanced C18:3n3 and C22:6n3 levels in Sanhuang chicken breast meat, suggesting potential health benefits for consumers. These improvements in PUFA content likely result from the synergistic modulation of lipid synthesis and oxidation by the probiotics and bioactive compounds in fermented ZSLs. However, HDG showed lower total n-3 PUFA and overall PUFA levels compared to MDG. This reduction may be due to the higher phenolic content in HDG, which could exert pro-oxidant activity and promote lipid peroxidation [34]. The pro-oxidant activity of phenolic compounds primarily occurs in the presence of transition metal ions or at elevated concentrations. Its core mechanism involves radical chain reactions and metal ion-mediated redox cycling, demonstrating that certain natural antioxidants also exhibit pro-oxidant effects [33].

### 3.5. Free Amino Acid Profile

Table 5 shows that the concentrations of five essential amino acids (EAA)—isoleucine, leucine, methionine, phenylalanine, and valine—were significantly higher in the group supplemented with fermented ZSLs compared to the control group (*p* < 0.05). Similarly, seven non-essential amino acids (NEAA), including alanine, arginine, aspartic acid, cysteine, glycine, proline, and serine, also increased significantly in the same group (*p* < 0.05). Notably, lysine was absent across all groups, while histidine was detected only in MDG. Overall, the inclusion of fermented ZSLs in the diet significantly enhanced total EAA, NEAA, and Umami amino acids (UAA) levels in Sanhuang chicken meat (*p* < 0.05). Additionally, UAA and sweet amino acids (SAAs) concentrations increased proportionally with the amount of fermented ZSLs in the diet (*p* < 0.05).

Amino acids, serving as the material foundation of protein metabolism, are not only of vital importance for biological functions but also key precursors in the formation of flavor compounds in meat. Feed composition is known to enhance chicken flavor, with appropriate supplementation further optimizing sensory characteristics [35]. Mohammed et al. [36] highlighted that elevated levels of total free amino acids enhance meat’s nutritional value. In this study, all treatment groups showed significant increases in total FAA, UAA, and SAA levels. Among the amino acids, glutamic acid was most prevalent, followed by threonine and alanine. Glutamic acid, a key precursor for glutathione synthesis, is crucial for antioxidant defense and is a primary umami compound, enhancing aroma and taste [37]. Umami amino acids also help reduce dietary salt and fat intake while increasing food consumption among the elderly [38]. In addition, the increased levels of SAA, such as alanine and glycine, may further enhance the sensory characteristics of chicken meat through synergistic interactions [35]. The improvement in amino acid composition by fermented ZSLs can be attributed to their abundant probiotics and bioactive compounds, which promote protein hydrolysis and inhibit amino acid oxidation, thereby increasing the release and accumulation of free amino acids. However, the increase in some amino acids was lower in HDG compared to MDG, suggesting that excessive addition may reduce amino acid synthesis efficiency due to increased metabolic load or substrate competition. Fermented ZSLs significantly enhanced the nutritional quality and flavor characteristics of chicken by regulating amino acid metabolism, with the 6% addition yielding the best results.

### 3.6. Sensory Assessment

**Figure 1:**
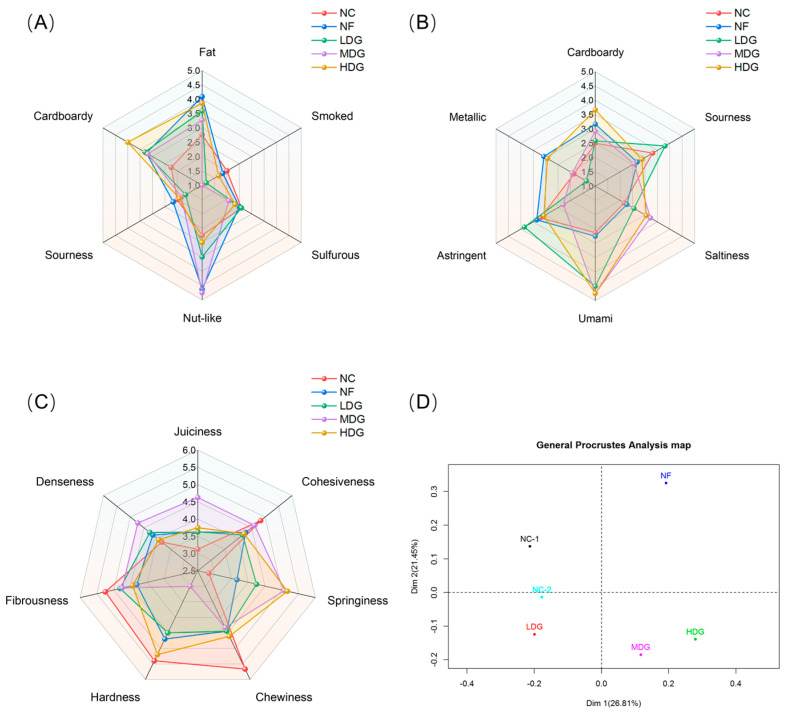
Sensory evaluation scores of Sanhuang chicken. (**A**) Flavor scores; (**B**) taste scores; (**C**) texture scores; (**D**) General Procrustes analysis (GPA) of the sensory data.

The sensory evaluation results of boiled chicken are illustrated in Figure 1. Compared with the control group, the dietary supplementation of fermented ZSLs improved the flavor, taste, and texture attributes of chicken. In terms of flavor, treatment groups showed significantly lower smoky flavor scores compared to the control group, while nutty flavors were more pronounced in the MDG and NF groups (Figure 1A). It is worth noting that the intensity of fat and cardboard flavors in the treatment groups was significantly higher than in the control group. This may be attributed to the accumulation of lipid oxidation products, such as aldehydes and ketones, particularly due to the oxidative degradation of PUFAs during processing or storage. This result indicates that while fermented Sichuan pepper leaves enhance the fatty acid composition (such as increasing n-3 PUFA), it is also essential to consider oxidative stability and shelf-life requirements.

In the sensory evaluation of taste, the MDG and HDG groups demonstrated higher scores for umami and saltiness (Figure 1B), which may be closely linked to the increased content of free amino acids. This study revealed that fermented ZSLs significantly elevated the levels of umami amino acids, such as glutamic acid and threonine (Table 5). Glutamic acid, the primary activator of umami receptors, can directly enhance the umami flavor of meat when its concentration rises. Furthermore, the improvement in saltiness is likely attributed to the synergistic flavor-enhancing effects of sodium ions and amino acids [30].

Texture analysis showed notable variation across attributes such as juiciness, elasticity, chewiness, and hardness (Figure 1C), with the MDG group scoring highest for juiciness and elasticity and lowest for hardness. This is thought to be attributed to the modification of the muscle fiber membrane structure and the enhancement of water-holding capacity by PUFAs. Although direct molecular evidence of PUFA–myofibrillar protein interactions is currently lacking, integrated analysis of lipidomic profiles, texture parameters, and sensory data suggests that PUFA enrichment likely enhances meat tenderness through improved myocyte membrane hydration capacity and the inhibition of collagen cross-linking in connective tissue. Generalized Procrustes analysis (GPA) based on sensory data (Figure 1D) further revealed a clear distinction in the sensory characteristics of chicken breast meat among the different treatment groups. In conclusion, dietary supplementation with ZSLs influenced the sensory qualities of Sanhuang chicken, improving flavor and overall acceptance. However, the specific reasons remain unclear, and further exploration through instrumental analysis is required.

### 3.7. E-Tongue and E-Nose

**Figure 2:**
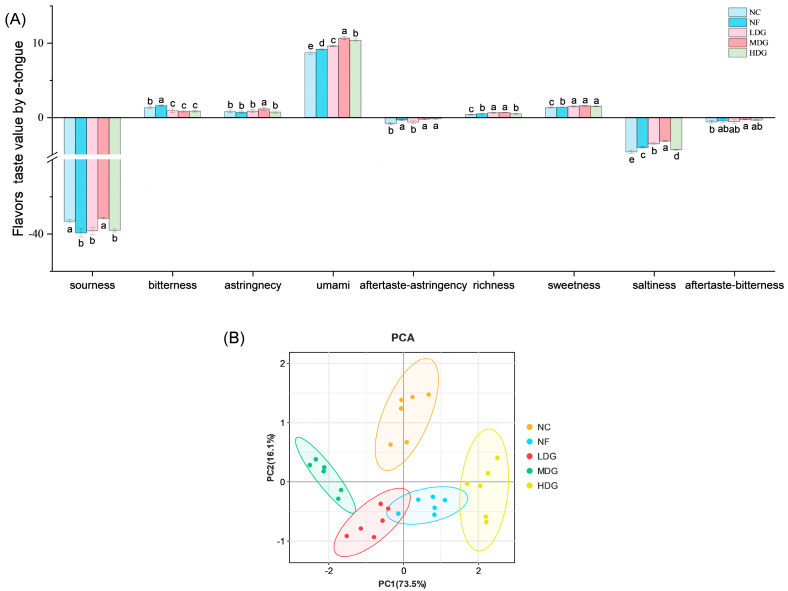
E-tongue analysis of Sanhuang chicken. (**A**) Flavor taste values by e-tongue; (**B**) principal component analysis (PCA) of e-tongue. Different lowercase letters indicate significant differences (*p* < 0.05).

To systematically investigate the impact of fermented ZSLs on the flavor profile of chicken breast meat, we employed e-tongue and e-nose technologies for quantitative analysis. These methods are recognized for their precision in characterizing flavor attributes [39]. The e-tongue measured parameters including sourness, sweetness, bitterness, aftertaste (bitterness), astringency, aftertaste (astringency), umami, saltiness, and richness. Using tasteless thresholds (sourness: −13; umami: −6; others: 0) as reference points. Meaningful responses were detected for bitterness, umami, richness, and sweetness, while sourness, aftertaste (bitterness), aftertaste (astringency), and saltiness remained consistently below the threshold across all groups (Figure 2A). The lack of responses may reflect the low concentration of flavor compounds in diluted liquid samples. The MDG group demonstrated significantly higher umami levels compared to the control group (*p* < 0.05). Richness was notably greater in the LDG and MDG groups (*p* < 0.05), and sweetness was significantly elevated in chickens fed with fermented ZSLs (*p* < 0.05). This result is closely linked to the increased content of FAA, particularly the elevated concentrations of glutamic acid (the primary precursor of umami), as well as alanine and glycine (sweet amino acids) [40]. This further supports the enhanced perception of umami and saltiness observed in the sensory evaluation (Figure 1B). The umami flavor formation theoretically relies on Glu-IMP synergism; however, instrumental constraints precluded quantification of IMP in this study. Principal component analysis (PCA) (Figure 2B) showed clear segregation between the control and treatment groups, indicating that the electronic tongue can effectively differentiate the flavor characteristics of the treatment group from those of the control group.

**Figure 3:**
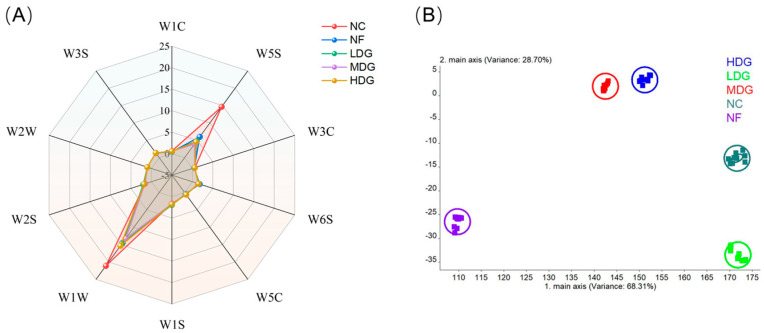
E-nose analysis of Sanhuang chicken. (**A**) Flavor taste values by e-nose; (**B**) linear discriminant analysis (LDA) of e-nose.

E-nose analysis further revealed the differences in volatile aromas (Figure 3). The result demonstrated that the chicken samples exhibited relatively high sensitivity to the W5S and W1W sensors, indicating greater concentrations of nitrogen oxides and organic sulfides in the chicken. Among all the treatment groups, the response values of W5S and W1W were significantly lower than those of the control group (Figure 3A), with the MDG group presenting the most significant decrease. The reduction in the response values of nitrogen oxides and organic sulfides indicates a decrease in unpleasant odors, contributing to a more balanced and milder flavor profile [41]. In other words, adding fermented ZSLs to the diet helps reduce unpleasant odors in chicken, with the MDG group showing the most significant effect. This result may be linked to the antioxidant activity of polyphenols in fermented ZSLs, which enhance the flavor of chicken by inhibiting lipid oxidation and the degradation of sulfur-containing amino acids (such as cysteine), while also reducing the formation of undesirable volatile compounds. Linear discriminant analysis (LDA) further confirmed the effective discrimination of the e-nose for each treatment group (Figure 3B), indicating that the addition of fermented ZSLs to the diet influences the formation of chicken breast meat flavor, as verified through both sensory and instrumental analysis. However, the specific volatile compounds responsible for these differences remain unclear, warranting further investigation to identify and compare key aroma compounds.

### 3.8. Volatile Compound Profiling

**Figure 4:**
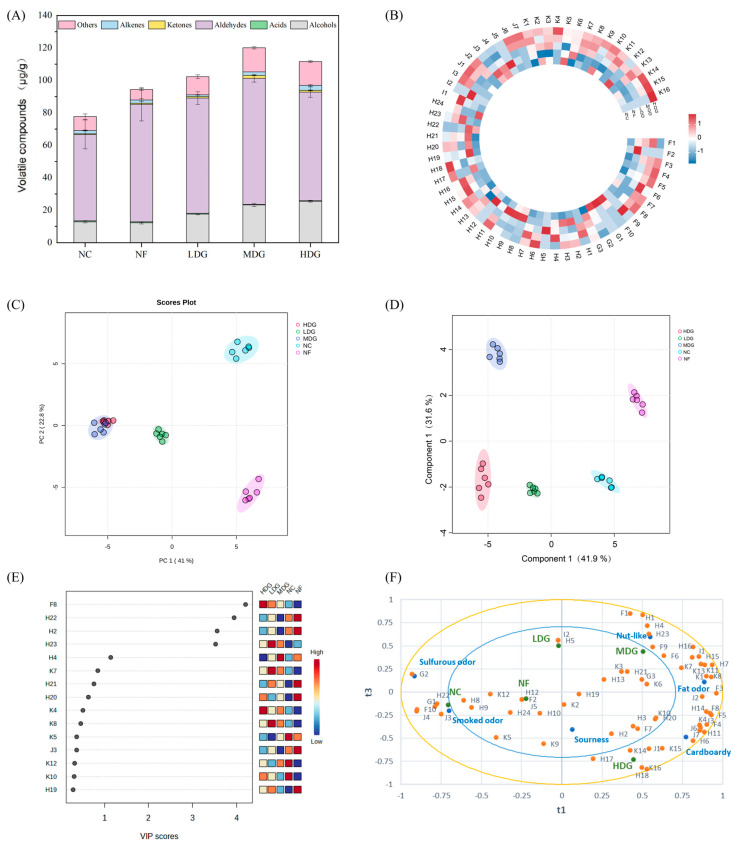
The profiles of volatile compounds of Sanhuang chicken based on GC-MS. (**A**) Statistical data on different categories of volatile compounds. (**B**) Heatmap of volatile compounds content of Sanhuang chicken; (**C**) PCA was used to compare the ability of GC-MS to distinguish chicken; (**D**) the Score Scatter Plot obtained by PLS-DA; (**E**) Variable Importance for Projection (VIP) scores plot of the PLS-DA model; (**F**) projection of PLS regression between aroma compounds and aroma attributes of chicken.

The volatile compounds in chicken were analyzed, with the results summarized in Table 6. GC-MS identified 63 volatile organic compounds across the samples, with 41, 48, 50, 53, and 52 compounds detected in the five chicken breast groups, respectively. The predominant volatile compounds included 1-octen-3-ol (3.49–5.22 µg/g), linalool (7.41–18.42 µg/g), hexanal (16.22–22.56 µg/g), benzaldehyde (4.28–10.91 µg/g), nonanal (9.32–13.85 µg/g), pentadecanal (2.33–8.09 µg/g), and hexadecanal (4.49–17.19 µg/g). Analysis of various compounds revealed that aldehydes were the predominant volatile compounds (53.15–77.78 µg/g), followed by alcohols (12.21–25.52 µg/g), with acids showing the lowest concentrations (Figure 4A). Fermented ZSL supplementation significantly increased the total aldehyde and alcohol content compared to the control group, with the highest total volatile compound content observed in MDG, followed by HDG. The heatmap (Figure 4B) indicates that the treatment groups, particularly MDG and HDG, displayed elevated concentrations and a greater diversity of volatile compounds compared to the control group. These results suggest that fermented ZSL supplementation enhances the abundance and variety of volatile compounds in chicken breast meat. This may be associated with the regulation of lipid and amino acid metabolism by probiotics, as well as enzymatic reactions during fermentation, which promote the oxidation and transformation of volatile precursor substances, such as unsaturated fatty acids and free amino acids [42].

Principal component analysis (PCA) and partial least squares discriminant analysis (PLS-DA) further highlighted the differences in the volatile substance composition between the control and treatment groups. Figure 4C shows the PCA score plot of volatile compound content measured by GC-MS, with the first two principal components accounting for 63.8% of the total variance. The treatment groups with fermented ZSLs are positioned on the left side of the PCA score plot, with MDG and HDG partially overlapping, indicating similar aroma profiles. In contrast, NC and NF are located farther from the other groups, reflecting that their volatile compound composition differs from the other samples. These results are consistent with findings from sensory and e-nose analyses. PCA effectively highlights classification trends and groups samples based on their volatile profiles. PLS-DA, a supervised discriminant analysis, was applied to correlate GC-MS-identified volatile compounds with sample categories [43]. As shown in Figure 4D, the first and second components account for 41.9% and 31.6% of the variance, respectively. The PLS-DA plot reveals clear separations among all five groups, with greater distances indicating larger differences in composition [40]. These findings confirm that GC-MS effectively discriminates between chicken samples based on their volatile compound profiles.

The supervised PLS-DA model was applied to identify aroma compounds with significant contributions to chicken sample classification and to pinpoint key differential aroma substances influencing chicken flavor. This approach evaluates the variable importance in projection (VIP) values, which measure the relevance and explanatory power of each volatile compound in distinguishing aroma profiles [44]. Compounds with VIP values above 1.0 are deemed critical for classification. As illustrated in Figure 4E, linalool, hexanal, benzaldehyde, pentadecanal, and hexadecanal all displayed VIP values exceeding 1.0, establishing them as key biomarkers for chicken flavor differentiation. Table 6 outlines the concentrations of these compounds across the sample groups. Among the five biomarkers, four (excluding hexanal) demonstrated significant differences between groups (*p* < 0.05). Therefore, linalool, benzaldehyde, pentadecanal, and hexadecanal were identified as the key differential compounds responsible for distinguishing the flavor characteristics of chicken. Fermented ZSL supplementation notably increased linalool levels, imparting floral and woody aromas. Benzaldehyde, recognized for its nutty scent, was significantly elevated in the NF, LDG, and MDG groups compared to NC and HDG, with MDG exhibiting the highest levels. This may be the primary reason for the intensified nut-like flavor observed in the sensory assessment of the MDG group. Furthermore, ZSL supplementation reduced pentadecanal, a compound associated with pungent and bitter flavor, which may explain the diminished bitterness in fermented groups [45]. In contrast, hexadecanal, known for its mild floral and waxy aroma, was more abundant in the treatment groups, contributing to a smoother, more refined chicken flavor profile.

PLSR analysis was used to explore the relationship between aroma compounds identified by GC-MS (X variables, *n* = 63) and six poultry aroma attributes evaluated through sensory analysis (Y variables, *n* = 6). As shown in Figure 4F, the weak sourness intensity may result from the limited presence of compounds near the acidic aroma zone. The NC sample was located closer to smoky and sulfurous aromas, while the treatment group was positioned farther away. This difference might be due to the higher content of aldehyde substances such as (E,E)-2,4-decadienal and nonanal in the treatment group, which produced stronger aldehyde-derived aromas and masked the smoky and sulfurous flavors. Ref. [46] The synergistic effect of benzaldehyde and hexadecanal enhances the nut-like aroma, resulting in a more mellow aroma of the chicken [47]. It is worth noting that compounds such as 1-octen-3-ol, with its broth-like scent, and (E,E)-2,4-decadienal, recognized for its rich chicken and chicken fat aromas, add a distinct chicken-like fragrance. Meanwhile, 1-octanol, nonanal, and (E)-2-octenal contribute oil or fried aroma, collectively enhancing the fat flavor profile of MDG and HDG. The cardboard-like aroma, associated with slightly oxidized fats and oils, evokes the impression of wet cardboard. The proximity of MDG and HDG to this aroma suggests a higher content of unsaturated fatty acids in these samples. During cooking, the oxidation and breakdown of unsaturated fatty acids generate aldehydes with oily characteristics, contributing to the unique flavor of cooked chicken [16]. Aldehyde compounds, characterized by high concentrations and diverse olfactory properties, play a key role in creating the overall chicken flavor.

## 4. Conclusions

This study reveals that dietary supplementation with fermented ZSLs significantly improved the growth performance, carcass traits, and meat quality of Sanhuang chickens. Supplementing 6% fermented ZSLs notably enhanced total PUFA levels, particularly n-3 PUFAs, which are recognized for their nutritional benefits. Furthermore, ZSL supplementation increased the levels of total FAA, UAA, and SAA, promoting consumer health while shaping the flavor profile of chicken meat. To investigate the effects of ZSLs on chicken flavor, both instrumental and sensory analyses were performed. Key aroma compounds identified included linalool, benzaldehyde, pentadecanal, and hexadecanal. Correlation analysis using the PLSR model linked sensory attributes to specific aroma compounds. For example, 1-octen-3-ol, (E,E)-2,4-decadienal, 1-octanol, and (E)-2-octenal showed positive associations with fat aroma and cardboard flavor, while benzaldehyde and hexadecanal were closely tied to a nutty flavor. Among the substitution levels tested, 6% fermented ZSLs produced the most significant improvements. In summary, the results highlight the potential of fermented ZSLs as a dietary supplement for improving the production and flavor quality of Sanhuang chickens. Modifying chicken flavor through fermented ZSLs is a promising approach that warrants further investigation.

## Figures and Tables

**Table 1:** Composition of ZSLs and fermented ZSLs.

Ingredients	ZSLs	Fermented ZSLs
Crude protein (%)	20.71 ± 0.17	19.16 ± 0.20
Acid-soluble protein (%)	1.36 ± 0.12	5.17 ± 0.11
Cellulose (%)	15.34 ± 0.18	9.39 ± 0.51
Crude ash (%)	7.56 ± 0.40	4.51 ± 0.26
Dry matter (g/kg)	882.90 ± 2.45	413.02 ± 2.55
Digestible energy (MJ/kg)	14.58 ± 0.18	16.44 ± 0.55
Lactic acid bacteria (log10CFU/g)	5.95 ± 0.06	8.53 ± 0.22
Yeast (log10CFU/g)	4.75 ± 0.21	4.83 ± 0.49
*Bacillus subtilis* (log10CFU/g)	7.69 ± 0.07	8.19 ± 0.07
Total phenols (mg·GAE/g)	91.55 ± 5.76	119.86 ± 5.65
Total flavonoids (mg·RE/g)	40.84 ± 1.42	100.93 ± 5.00

The data are expressed as the mean ± SD (*n* = 3).

**Table 2:** The growth performance and carcass characteristics of Sanhuang chickens.

Items	Treatment				
	NC	NF	LDG	MDG	HDG
Growth performance					
ABW1, g	46.47 ± 0.88 a	46.60 ± 0.83 a	46.58 ± 0.73 a	46.81 ± 0.65 a	47.12 ± 0.23 a
ABW2, kg	1.53 ± 0.11 d	1.71 ± 0.05 c	1.65 ± 0.09 cd	1.98 ± 0.08 a	1.84 ± 0.14 b
ADG, g/d	21.12 ± 1.47 d	23.74 ± 0.76 c	22.91 ± 0.20 cd	27.66 ± 1.14 a	25.64 ± 1.99 b
ADFI, g/d	70.38 ± 2.76 d	76.43 ± 0.99 c	76.92 ± 0.85 c	83.93 ± 1.10 a	81.12 ± 0.74 b
F: G	3.35 ± 0.29 a	3.22 ± 0.14 ab	3.37 ± 0.17 a	3.04 ± 0.14 b	3.18 ± 0.26 ab
Carcass traits					
DP, %	87.03 ± 1.98 b	87.73 ± 1.66 ab	89.43 ± 1.92 a	88.61 ± 1.15 ab	88.59 ± 0.83 ab
SEP, %	79.45 ± 2.07 a	79.42 ± 1.31 a	81.57 ± 1.70 a	80.24 ± 1.68 a	80.64 ± 0.69 a
EP, %	63.62 ± 1.89 a	63.69 ± 1.69 a	64.24 ± 1.42 a	64.77 ± 1.51 a	64.43 ± 3.58 a
BMP, %	17.32 ± 1.61 a	17.73 ± 1.35 a	17.78 ± 1.68 a	17.82 ± 2.51 a	18.24 ± 1.40 a
LMP, %	20.44 ± 1.31 a	20.72 ± 1.34 a	20.85 ± 2.75 a	21.95 ± 3.40 a	21.55 ± 1.42 a
SFT, cm	0.41 ± 0.07 a	0.44 ± 0.05 a	0.42 ± 0.08 a	0.46 ± 0.06 a	0.45 ± 0.06 a
AFP, %	2.87 ± 0.41 b	3.29 ± 0.39 ab	3.62 ± 0.35 a	2.22 ± 0.34 c	2.74 ± 0.64 bc

The data are expressed as the mean ± SD (*n* = 6). Mean values with different letters were considered significantly different at *p* < 0.05. NC, a basal diet; NF, a basal diet supplemented with 6% unfermented ZSL; LDG, a basal diet supplemented with 3% fermented ZSL; MDG, a basal diet supplemented with 6% fermented ZSL; HDG, a basal diet supplemented with 9% fermented ZSL. ABW1, average body weight on day 1; ABW2, average body weight on day 71; ADG, average daily gain; ADFI, average daily feed intake; F: G, feed-to-gain ratio was computed by dividing ADFI by ADG; DP, dressing percentage; SEP, semi-eviscerated percentage; EP, eviscerated percentage; BMP, breast muscle percentage; LMP, leg muscle percentage; SFT, subcutaneous fat thickness; AFP, abdominal fat percentage.

**Table 3:** The raw meat quality of Sanhuang chickens.

Items	Treatment				
	NC	NF	LDG	MDG	HDG
IMF, g/100 g	0.71 ± 0.09 b	0.78 ± 0.07 ab	0.77 ± 0.04 ab	0.84 ± 0.03 a	0.83 ± 0.04 a
MP, g/100 g	24.94 ± 0.26 a	24.80 ± 0.60 a	24.92 ± 0.33 a	24.75 ± 0.48 a	24.86 ± 0.51 a
pH 45 min	6.13 ± 0.05 a	6.12 ± 0.04 a	6.13 ± 0.04 a	6.15 ± 0.03 a	6.15 ± 0.04 a
pH 24 h	5.76 ± 0.03 c	5.78 ± 0.02 bc	5.83 ± 0.03 b	6.01 ± 0.07 a	6.06 ± 0.05 a
L* 24 h	51.70 ± 0.53 a	52.51 ± 0.58 a	51.40 ± 0.29 a	50.53 ± 0.29 a	50.13 ± 0.09 a
a* 24 h	3.96 ± 0.27 a	3.60 ± 0.30 b	3.17 ± 0.08 c	3.27 ± 0.07 c	3.51 ± 0.11 b
b* 24 h	14.24 ± 0.50 a	13.67 ± 0.60 ab	13.73 ± 0.76 ab	12.88 ± 0.77 bc	12.42 ± 0.51 c
Cooking loss rate, %	29.70 ± 1.70 a	28.83 ± 2.28 ab	28.93 ± 1.81 ab	27.94 ± 1.73 ab	27.04 ± 1.23 b
Drip loss rate, %	5.35 ± 0.47 a	4.69 ± 0.49 ab	4.12 ± 0.63 bc	4.02 ± 0.64 bc	3.90 ± 0.39 c
Shear force, N	16.5 ± 1.22 a	14.08 ± 0.71 b	11.03 ± 0.29 c	10.47 ± 1.49 cd	9.17 ± 0.97 d

The data are expressed as the mean ± SD (*n* = 6). Mean values with different letters were considered significantly different at *p* < 0.05. NC, a basal diet; NF, a basal diet supplemented with 6% unfermented ZSLs; LDG, a basal diet supplemented with 3% fermented ZSLs; MDG, a basal diet supplemented with 6% fermented ZSLs; HDG, a basal diet supplemented with 9% fermented ZSLs. IMF, intramuscular fat; MP, muscle protein. L*, lightness; a*, redness; b*, yellowness.

**Table 4:** The fatty acid composition (mg/100 g tissue, dry matter basis) in the raw meat of Sanhuang chicken.

Items	Treatment				
	NC	NF	LDG	MDG	HDG
C14:0	23.25 ± 0.73 b	28.49 ± 3.57 a	16.70 ± 1.20 c	26.25 ± 2.81 ab	21.96 ± 1.73 b
C14:1	3.77 ± 0.36 b	5.11 ± 0.98 a	4.42 ± 0.57 ab	4.44 ± 0.28 ab	4.11 ± 0.24 ab
C16:0	312.44 ± 4.30 a	296.36 ± 3.65 c	304.60 ± 7.07 b	291.72 ± 2.91 c	293.87 ± 1.91 c
C16:1	8.92 ± 0.05 a	7.46 ± 0.05 b	7.32 ± 0.12 b	5.97 ± 0.02 c	7.34 ± 0.13 b
C18:0	103.11 ± 0.41 c	104.88 ± 0.58 c	110.62 ± 1.33 a	111.53 ± 0.90 a	108.11 ± 1.42 b
C18:1n9t	209.95 ± 0.17	207.81 ± 2.10	211.26 ± 4.45	209.67 ± 0.57	210.42 ± 2.25
C18:2n6c	260.00 ± 7.18 a	250.16 ± 0.41 c	240.99 ± 1.21 d	252.91 ± 0.51 bc	258.53 ± 2.91 ab
C18:3n3	22.04 ± 0.73 b	30.06 ± 1.85 a	17.48 ± 1.09 b	29.61 ± 2.35 a	21.96 ± 2.34 b
C20:2	2.00 ± 0.18 d	3.78 ± 0.26 bc	3.30 ± 0.28 c	4.54 ± 0.25 a	3.94 ± 0.39 b
C20:3n6	18.68 ± 1.81	17.55 ± 4.46	18.35 ± 1.83	17.77 ± 2.46	17.65 ± 0.38
C20:4n6	63.67 ± 4.22 a	55.78 ± 0.41 ab	54.47 ± 6.55 ab	44.29 ± 0.59 b	43.85 ± 1.13 b
C22:6n3	21.56 ± 1.94 c	31.19 ± 3.01 b	39.87 ± 1.42 a	43.64 ± 1.71 a	40.41 ± 1.91 a
SFA	438.80 ± 1.34 a	429.73 ± 3.71 b	431.92 ± 5.73 b	429.51 ± 0.98 b	426.95 ± 4.65 b
MUFA	222.63 ± 0.42 b	220.38 ± 1.75 b	227.03 ± 0.85 a	220.07 ± 0.69 b	221.87 ± 2.38 b
PUFA	385.96 ± 6.92 b	384.74 ± 3.17 b	371.17 ± 8.34 b	388.23 ± 0.37 a	383.40 ± 0.76 b
Σn-6 PUFA	323.67 ± 10.95 a	305.94 ± 0.23 b	295.47 ± 7.50 b	297.20 ± 0.81 b	302.38 ± 0.86 b
Σn-3 PUFA	62.29 ± 4.03 c	78.79 ± 3.08 b	75.70 ± 6.82 b	91.02 ± 1.16 a	80.02 ± 1.75 b

The data are expressed as the mean ± SD (*n* = 6). Mean values with different letters were considered significantly different at *p* < 0.05. NC, a basal diet; NF, a basal diet supplemented with 6% unfermented ZSLs; LDG, a basal diet supplemented with 3% fermented ZSLs; MDG, a basal diet supplemented with 6% fermented ZSLs; HDG, a basal diet supplemented with 9% fermented ZSLs. SFAs, saturated fatty acids; MUFAs, monounsaturated fatty acids; PUFAs, polyunsaturated fatty acids.

**Table 5:** Free amino acid composition (mg/100 g tissue, dry matter basis) in the raw meat of Sanhuang chicken.

Items	Treatment				
	NC	NF	LDG	MDG	HDG
Essential amino acids					
Histidine	ND	ND	ND	42.36 ± 0.91	ND
Isoleucine	9.05 ± 0.14 c	8.80 ± 0.26 c	11.22 ± 0.19 b	11.61 ± 0.26 a	11.12 ± 0.37 b
Leucine	15.39 ± 0.39 d	14.90 ± 0.57 d	19.81 ± 0.63 b	22.93 ± 0.64 a	19.14 ± 0.21 c
Lysine	ND	ND	ND	ND	ND
Methionine	7.27 ± 0.13 e	8.29 ± 0.21 d	9.85 ± 0.11 c	11.52 ± 0.49 a	11.12 ± 0.18 b
Phenylalanine	15.69 ± 0.53 c	14.13 ± 0.35 d	17.25 ± 0.70 b	21.38 ± 0.67 a	16.90 ± 0.53 b
Threonine	35.45 ± 0.92 c	34.28 ± 0.99 d	38.73 ± 0.38 b	31.54 ± 0.59 e	40.25 ± 0.87 a
Valine	13.40 ± 0.38 c	14.50 ± 0.42 b	17.58 ± 0.74 a	17.41 ± 0.28 a	17.67 ± 0.24 a
Non-essential amino acids					
Alanine	15.31 ± 0.44 d	17.11 ± 0.42 c	19.51 ± 0.72 b	19.30 ± 0.54 b	20.82 ± 0.74 a
Arginine	15.31 ± 0.44 d	17.11 ± 0.42 c	19.51 ± 0.72 b	19.30 ± 0.54 b	20.82 ± 0.74 a
Aspartic acid	6.14 ± 1.66 e	8.09 ± 0.79 d	10.04 ± 0.21 c	12.14 ± 0.35 a	11.52 ± 0.49 b
Cysteine	2.54 ± 0.21 d	3.00 ± 0.14 c	3.18 ± 0.14 c	7.23 ± 0.54 a	4.05 ± 0.33 b
Glutamic acid	43.24 ± 0.88 a	48.49 ± 0.61 b	52.84 ± 0.58 c	53.35 ± 0.49 d	55.86 ± 1.00 e
Glycine	17.09 ± 0.53 d	20.72 ± 1.34 d	20.44 ± 1.31 c	21.95 ± 3.40 a	21.55 ± 1.42 b
Proline	24.22 ± 0.62 c	26.93 ± 0.83 d	26.50 ± 1.14 b	26.98 ± 1.21 b	29.54 ± 0.73 a
Serine	29.49 ± 0.74 d	28.43 ± 0.80 e	31.47 ± 1.09 c	36.40 ± 0.79 b	40.05 ± 0.55 a
Tyrosine	12.71 ± 0.28 a	12.03 ± 0.37 b	13.05 ± 0.39 a	11.56 ± 0.37 c	11.83 ± 0.16 bc
Total EAA	96.24 ± 1.40 d	94.90 ± 1.21 d	114.43 ± 1.42 c	158.74 ± 1.43 a	116.19 ± 1.22 b
Total NEAA	192.90 ± 1.21 e	199.49 ± 0.81 d	215.64 ± 2.39 c	230.94 ± 2.24 b	237.63 ± 1.84 a
Total FAA	289.14 ± 1.54 d	294.39 ± 1.44 d	330.07 ± 2.99 c	389.68 ± 2.81 a	353.82 ± 2.60 b
UAA ^1^	49.38 ± 1.02 e	56.57 ± 0.63 d	62.87 ± 0.64 c	65.49 ± 0.64 b	67.38 ± 1.44 a
SAA ^2^	88.74 ± 0.95 d	83.85 ± 0.91 e	90.51 ± 1.22 c	100.39 ± 1.04 b	104.02 ± 0.31 a
EAA/NEAA	0.50 ± 0.01 c	0.48 ± 0.01 e	0.53 ± 0.01 b	0.69 ± 0.01 a	0.49 ± 0.00 d

The data are expressed as the mean ± SD (*n* = 6). Mean values with different letters were considered significantly different at *p* < 0.05. NC, a basal diet; NF, a basal diet supplemented with 6% unfermented ZSLs; LDG, a basal diet supplemented with 3% fermented ZSLs; MDG, a basal diet supplemented with 6% fermented ZSLs; HDG, a basal diet supplemented with 9% fermented ZSLs. ND denotes not detected. EAAs, essential amino acids; NEAAs, non-essential amino acids; FAAs, free amino acids; UAAs, umami amino acids; SAAs, sweet amino acids. ^1^ Umami amino acids = aspartic acid + glutamic acid; ^2^ sweet amino acids = alanine + glycine + serine.

**Table 6:** The volatile compounds (µg/g tissue, wet fresh matter basis) in boiled chicken breast.

No.	Volatile Compounds	RT	RI	CAS	Treatment				
					NC	NF	LDG	MDG	HDG
F1	1-Pentanol	4.67	765	71-41-0	ND	0.22 ± 0.03	0.28 ± 0.04	0.41 ± 0.04	ND
F2	1-Hexanol	8.18	868	111-27-3	ND	0.15 ± 0.04	ND	ND	ND
F3	1-Heptanol	12.73	970	111-70-6	ND	0.08 ± 0.02	0.19 ± 0.06	0.22 ± 0.02	0.25 ± 0.02
F4	1-Octen-3-ol	13.11	980	3391-86-4	3.49 ± 0.26	3.58 ± 0.26	3.94 ± 0.25	4.68 ± 0.04	5.22 ± 0.28
F5	2-ethyl-1-Hexanol	15.34	1030	104-76-7	ND	0.19 ± 0.05	0.21 ± 0.06	0.27 ± 0.04	0.39 ± 0.05
F6	(E)-2-Octen-1-ol	16.93	1060	18409-17-1	0.59 ± 0.04	0.44 ± 0.05	0.61 ± 0.04	1.03 ± 0.18	0.65 ± 0.20
F7	1-Octanol	17.04	1070	111-87-5	0.39 ± 0.10	ND	ND	0.58 ± 0.09	0.60 ± 0.06
F8	Linalool	18.08	1099	78-70-6	7.90 ± 0.12 ^d^	7.41 ± 0.82 ^d^	12.40 ± 0.79 ^c^	15.65 ± 0.87 ^b^	18.42 ± 1.07 ^a^
F9	Terpinen-4-ol	20.71	1182	562-74-3	ND	ND	ND	0.34 ± 0.09	ND
F10	1-Dodecanol	29.07	1474	112-53-8	0.42 ± 0.24	0.15 ± 0.03	ND	ND	ND
	**Alcohols**				12.79 ± 0.54 ^d^	12.22 ± 0.70 ^d^	17.63 ± 0.50 ^c^	23.19 ± 0.97 ^b^	25.53 ± 0.58 ^a^
G1	Nonanoic acid	23.64	1273	112-05-0	0.22 ± 0.04	ND	ND	ND	ND
G2	Hexadecanoic acid	38.75	1963	1957-10-3	0.47 ± 0.09	0.21 ± 0.06	0.19 ± 0.01	0.12 ± 0.01	ND
G3	Linoleic acid	40.81	2141	60-33-3	ND	0.34 ± 0.04	0.28 ± 0.02	0.21 ± 0.05	0.24 ± 0.04
	**Acids**				0.69 ± 0.10 ^a^	0.56 ± 0.10 ^ab^	0.47 ± 0.02 ^bc^	0.33 ± 0.05 ^d^	0.24 ± 0.06 ^d^
H1	Pentanal	3.14	700	110-62-3	ND	ND	0.74 ± 0.20	0.98 ± 0.42	ND
H2	Hexanal	5.54	801	66-25-1	16.22 ± 5.80 ^a^	22.56 ± 4.19 ^a^	16.27 ± 2.05 ^a^	18.63 ± 2.33 ^a^	20.47 ± 1.75 ^a^
H3	Heptanal	9.45	901	111-71-7	0.64 ± 0.12	0.93 ± 0.18	0.73 ± 0.09	0.77 ± 0.33	0.87 ± 0.08
H4	Benzaldehyde	12.10	962	100-52-7	4.28 ± 0.18 ^c^	7.18 ± 0.58 ^b^	7.07 ± 0.27 ^b^	10.91 ± 0.04 ^a^	4.67 ± 0.10 ^c^
H5	Benzeneacetaldehyde	15.87	1045	122-78-1	ND	ND	0.10 ± 0.01	ND	ND
H6	(E)-2-Octenal	16.47	1060	2548-87-0	0.48 ± 0.05	0.50 ± 0.06	0.56 ± 0.04	0.76 ± 0.12	1.04 ± 0.05
H7	Nonanal	18.23	1104	124-19-6	9.32 ± 1.08	10.18 ± 1.19	11.57 ± 0.35	13.85 ± 0.71	12.08 ± 0.43
H8	(E)-2-Nonenal	20.15	1175	18829-56-6	0.32 ± 0.04	0.49 ± 0.03	0.22 ± 0.01	0.21 ± 0.03	0.19 ± 0.03
H9	3-ethyl-Benzaldehyde	20.22	1168	34246-54-3	0.26 ± 0.08	0.48 ± 0.04	0.18 ± 0.01	0.13 ± 0.02	0.17 ± 0.03
H10	(E)-4-Decenal	21.28	1193	21,662-09-9	0.33 ± 0.07	0.54 ± 0.06	0.37 ± 0.06	0.33 ± 0.05	0.39 ± 0.02
H11	Decanal	21.65	1206	112-31-2	1.34 ± 0.13	1.61 ± 0.07	1.47 ± 0.08	1.78 ± 0.07	1.96 ± 0.05
H12	(E,E)-2,4-Nonadienal	21.90	1216	5910-87-2	ND	0.16 ± 0.02	ND	ND	ND
H13	(E)-2-Decenal	23.34	1263	3913-81-3	0.56 ± 0.09	ND	0.69 ± 0.10	0.57 ± 0.01	0.60 ± 0.07
H14	Undecanal	24.67	1307	112-44-7	0.35 ± 0.04	0.47 ± 0.03	0.42 ± 0.02	0.52 ± 0.09	0.53 ± 0.04
H15	(E,E)-2,4-Decadienal	24.93	1317	25152-84-5	0.37 ± 0.05	0.46 ± 0.09	0.79 ± 0.26	1.49 ± 0.11	0.86 ± 0.17
H16	(E)-2-Undecenal	26.23	1365	53448-07-0	0.69 ± 0.05	0.71 ± 0.07	1.17 ± 0.17	1.56 ± 0.04	1.06 ± 0.09
H17	(E)-2-butyloct-2-enal	26.50	1378	13019-16-4	ND	0.19 ± 0.06	ND	ND	0.18 ± 0.03
H18	Dodecanal	27.42	1409	112-54-9	1.52 ± 0.13	1.59 ± 0.39	1.30 ± 0.25	1.65 ± 0.17	2.19 ± 0.20
H19	4-pentyl-Benzaldehyde	28.75	1476	6853-57-2	ND	1.05 ± 0.07	0.39 ± 0.05	0.30 ± 0.04	0.38 ± 0.07
H20	Tridecanal	29.99	1512	10486-19-8	2.67 ± 0.36	4.47 ± 0.81	2.93 ± 0.35	4.05 ± 0.32	4.11 ± 0.52
H21	Tetradecanal	32.39	1613	124-25-4	2.47 ± 1.38	4.92 ± 1.09	3.87 ± 0.13	4.03 ± 0.24	3.60 ± 0.69
H22	Pentadecanal	34.67	1715	2765-11-9	6.31 ± 1.88 ^a^	8.09 ± 1.79 ^a^	2.98 ± 0.36 ^b^	2.33 ± 0.06 ^b^	2.37 ± 0.07 ^b^
H23	Hexadecanal	36.65	1817	629-80-1	4.49 ± 0.42 ^d^	5.87 ± 1.90 ^cd^	17.19 ± 2.51 ^a^	12.67 ± 1.40 ^b^	9.05 ± 2.44 ^bc^
H24	9-Octadecenal	39.22	1997	5090-41-5	0.53 ± 0.03	ND	ND	0.28 ± 0.08	0.20 ± 0.07
	**Aldehydes**				53.15 ± 8.90 ^b^	72.44 ± 10.52 ^a^	71.00 ± 3.39 ^a^	77.78 ± 2.37 ^a^	66.96 ± 3.22 ^ab^
I1	2-Octanone	13.63	991	111-13-7	0.51 ± 0.01	0.67 ± 0.06	0.76 ± 0.04	1.52 ± 0.14	0.87 ± 0.23
I2	Acetophenone	16.77	1066	98-86-2	ND	ND	0.09 ± 0.01	ND	ND
I3	Geranylacetone	28.56	1453	3796-70-1	ND	ND	ND	0.22 ± 0.02	0.26 ± 0.04
	**Ketones**				0.51 ± 0.01 ^d^	0.67 ± 0.06 ^cd^	0.85 ± 0.05 ^bc^	1.74 ± 0.16 ^a^	1.13 ± 0.23 ^b^
J1	D-Limonene	15.16	1031	5989-27-5	0.96 ± 0.16	0.50 ± 0.04	0.42 ± 0.04	1.23 ± 0.03	1.66 ± 0.21
J2	1-Tetradecene	26.96	1392	1120-36-1	ND	ND	0.17 ± 0.04	0.17 ± 0.01	0.23 ± 0.02
J3	β-Caryophyllene	27.76	1419	87-44-5	0.63 ± 0.36	0.87 ± 0.40	0.36 ± 0.13	0.17 ± 0.05	0.33 ± 0.27
J4	α-Humulene	28.65	1454	6753-98-6	0.37 ± 0.10	0.39 ± 0.20	0.19 ± 0.11	ND	0.12 ± 0.06
J5	β-selinene	29.48	1486	17066-67-0	ND	0.11 ± 0.09	ND	0.00	0.00
J6	1-Pentadecene	29.52	1492	13360-61-7	ND	0.07 ± 0.01	ND	0.43 ± 0.04	0.50 ± 0.04
J7	Cetene	31.91	1592	629-73-2	ND	ND	ND	0.17 ± 0.02	0.21 ± 0.03
	**Alkenes**				1.96 ± 0.30 ^bc^	1.94 ± 0.75 ^bc^	1.13 ± 0.17 ^c^	2.17 ± 0.05 ^ab^	3.05 ± 0.48 ^a^
K1	2-pentyl-Furan	13.61	993	3777-69-3	ND	1.72 ± 0.03	1.76 ± 0.04	2.51 ± 0.05	2.12 ± 0.16
K2	Naphthalene	20.82	1182	91-20-3	0.22 ± 0.01	ND	0.20 ± 0.03	0.14 ± 0.01	0.21 ± 0.04
K3	Dodecane	21.46	1200	112-40-3	0.70 ± 0.12	0.21 ± 0.04	0.91 ± 0.13	0.82 ± 0.08	0.79 ± 0.08
K4	1,3-Di-tert-butylbenzene	23.13	1249	1014-60-4	0.20 ± 0.02	0.20 ± 0.04	0.49 ± 0.30	0.94 ± 0.07	1.46 ± 0.11
K5	Anethole	24.05	1287	104-46-1	1.38 ± 0.31	0.88 ± 0.09	0.32 ± 0.15	0.91 ± 0.02	0.92 ± 0.24
K6	2-n-Octylfuran	24.29	1297	4179-38-8	ND	0.60 ± 0.05	0.38 ± 0.04	0.39 ± 0.03	0.39 ± 0.02
K7	Tridecane	24.45	1300	629-50-5	1.46 ± 0.27	0.79 ± 0.05	2.67 ± 0.32	2.93 ± 0.22	2.58 ± 0.08
K8	1-ethylidene-1H-Indene	24.71	1315	2471-83-2	ND	ND	0.84 ± 0.13	1.09 ± 0.00	0.98 ± 0.04
K9	3-methyl-Tridecane	26.38	1371	6418-41-3	0.25 ± 0.02	0.12 ± 0.03	0.17 ± 0.03	0.18 ± 0.01	0.24 ± 0.01
K10	Tetradecane	27.17	1400	629-59-4	1.54 ± 0.23	0.84 ± 0.13	1.43 ± 0.26	1.82 ± 0.13	2.07 ± 0.04
K11	1,3-dimethyl-Naphthalene	27.66	1417	575-41-7	ND	ND	0.35 ± 0.06	0.51 ± 0.07	0.36 ± 0.06
K12	Pentadecane	29.69	1500	629-62-9	1.82 ± 0.48	0.42 ± 0.07	0.45 ± 0.04	1.14 ± 0.10	0.64 ± 0.31
K13	n-Nonylcyclohexane	30.96	1556	2883-02-5	0.17 ± 0.13	0.15 ± 0.11	0.41 ± 0.13	0.51 ± 0.04	0.41 ± 0.06
K14	3-methyl-Pentadecane	31.40	1570	2882-96-4	0.22 ± 0.05	0.10 ± 0.08	0.15 ± 0.01	0.21 ± 0.02	0.32 ± 0.03
K15	Hexadecane	32.09	1600	544-76-3	0.64 ± 0.04	0.50 ± 0.23	0.59 ± 0.05	0.72 ± 0.05	0.95 ± 0.16
K16	Heptadecane	34.33	1700	629-78-7	ND	ND	ND	ND	0.23 ± 0.08
	**Others**				8.59 ± 1.70 ^c^	6.54 ± 0.57 ^cd^	11.13 ± 1.21 ^b^	14.82 ± 0.71 ^a^	14.67 ± 0.56 ^a^

The data are expressed as the mean ± SD (*n* = 6). Mean values with different letters were considered significantly different at *p* < 0.05. NC, a basal diet; NF, a basal diet supplemented with 6% unfermented ZSLs; LDG, a basal diet supplemented with 3% fermented ZSLs; MDG, a basal diet supplemented with 6% fermented ZSLs; HDG, a basal diet supplemented with 9% fermented ZSLs. ND denotes not detected.

## Data Availability

Data will be made available on request from the corresponding author. The data are not publicly available due to privacy restrictions.

## Supplementary Materials

The following supporting information can be downloaded at https://www.mdpi.com/article/10.3390/foods14142542/s1, Figure S1: Differential metabolites before and after fermentation. (**A**) Positive ion mode; (**B**) negative ion mode; Table S1: Composition and nutrient levels of basal diet; Table S2: Sensory descriptors and definitions for Sanhuang chicken; institutional ethics guidelines.

## Abbreviations

The following abbreviations are used in this manuscript:

ABW_1_	average body weight on day 1
ABW_2_	average body weight on day 71
ADG	average daily gain
ADFI	average daily feed intake
SEP	semi-eviscerated percentage
EP	eviscerated percentage
BMP	breast muscle percentage
LMP	leg muscle percentage
SFT	subcutaneous fat thickness
AFP	abdominal fat percentage
IMF	intramuscular fat
MP	muscle protein
SFAs	saturated fatty acids
MUFAs	monounsaturated fatty acids
PUFAs	polyunsaturated fatty acids
EAAs	essential amino acids
NEAAs	non-essential amino acids
FAAs	free amino acids
SAAs	sweet amino acids
UAAs	umami amino acids
