# Supplementary material for: Effects of Dietary Supplementation with Fermented Zanthoxylum schinifolium Leaves on Growth Performance, Meat Quality, and Sensory Traits in Sanhuang Chicken
Source: Foods. 2025 Jul 21;14(14):2542. doi: 10.3390/foods14142542 (PMC12295701; doi:10.3390/foods14142542)
Supplement: Supplementary file 1 [file foods-14-02542-s001.zip › foods-3742323-supplementary.pdf]

Table. S1. Composition and nutrient levels of basal diet

**Ingredients** (%)	**starter (0-30d)**	**grower (31-71d)**
Corn	56.45	58.32
Soybean meal	33.41	30.18
Fish meal	3.00	2.67
Soybean oil	2.06	3.65
Limestone	0.87	1.13
CaHPO4	1.26	0.91
NaCl	0.30	0.30
L-Lysine	0.87	0.64
DL-Methionine	0.78	1.20
Premix1	1.00	1.00
Total	100	100
Nutrient levels		
Metabolizable energy (MJ/kg)	12.75	12.97
Crude protein	20.14	15.56
Crude fiber	5.00	6.70
Crude Ash	6.82	7.32
Calcium	0.81	0.89

[1]The premix provided per kg of diets: VA 10000 IU, VD 1000 IU, VE 500 mg, VK 200 mg, VB_1 100 mg, VB_2 200 mg, VB_6 100 mg, VB_{12} 15 mg, Nicotinic acid 0.2 g, Pantothenic acid 50 mg, Folic acid 20 mg, Biotin 0.5 mg, Cu 10 mg, Fe 100 mg, Zn 70 mg, Mn 55 mg, Se 0.35 mg, I 0.3 mg.

Table. S2. Sensory descriptors and definitions for Sanhuang chicken

Sensory types	Descriptors	Definition	Reference control samples
Texture	Juiciness	The moisture content in the sample	Hawthorn = 3; Cucumber = 7
	Cohesiveness	The degree to which chewed samples remain stuck together in the mouth	Steamed Rice Cake = 3
	Springiness	The degree to which the first bite sample regains its original shape after partial compression	Ham sausage = 4.5
	Chewiness	The number of times a sample is chewed until it is swallowed (or spit out)	Bread = 4.5; Beef Jerky = 8
	Hardness	The force exerted by the molars on the sample during the first two bites	Ham sausage = 3; Peanuts = 7
	Fibrousness	The filamentous structures felt by the tongue while chewing the sample	Parsley = 5; Celery = 7.5
	Denseness	The crushing degree of the cross-sectional area of the bite sample	Bread = 3; Ham sausage = 6
Flavor	Fat odor	The aroma is associated with cooked chicken fat	Chicken fat = 5
	Smoked odor	Aroma related to smoke	1 drop smoke-infused essence dissolved in 150 mL water = 4.5
	Sulfurous odor	A smell associated with rotten eggs	2.5 g cooked and mashed chicken egg whites = 4
	Nut-like	A nutty flavor similar to that of nuts	Five ripe almonds = 5.5
	Sourness	A pungent odor similar to vinegar	4% Glutinous Rice White Vinegar = 5.5
	Cardboardy	It is associated with slightly oxidized fats and oils, reminiscent of wet cardboard packaging	1/2 slice of Aji soda Cracker = 3; 1 Aji cracker = 4.5

Taste	Cardboardy	It is associated with slightly oxidized fats and oils, reminiscent of wet cardboard packaging	1/3 slice of Aji Cracker = 7
	Sourness	The basic taste caused by acid	0.05g/L anhydrous citric acid
	Saltiness	The taste of sodium chloride water solution	0.15% sodium chloride solution = 2.5
	Umami	The chemical sensation is caused by umami amino acids	0.1% Monosodium glutamate = 3
	Astringent	The constricting sensation caused by the protein in the oral mucosa being solidified	0.03% food alum = 2; 0.04% food alum = 5
	Metallic	This refers to the taste sensation produced by metal ions stimulating the taste receptors	0.05% FeSO4·7H2O=5; 0.1% FeSO4·7H2O=7

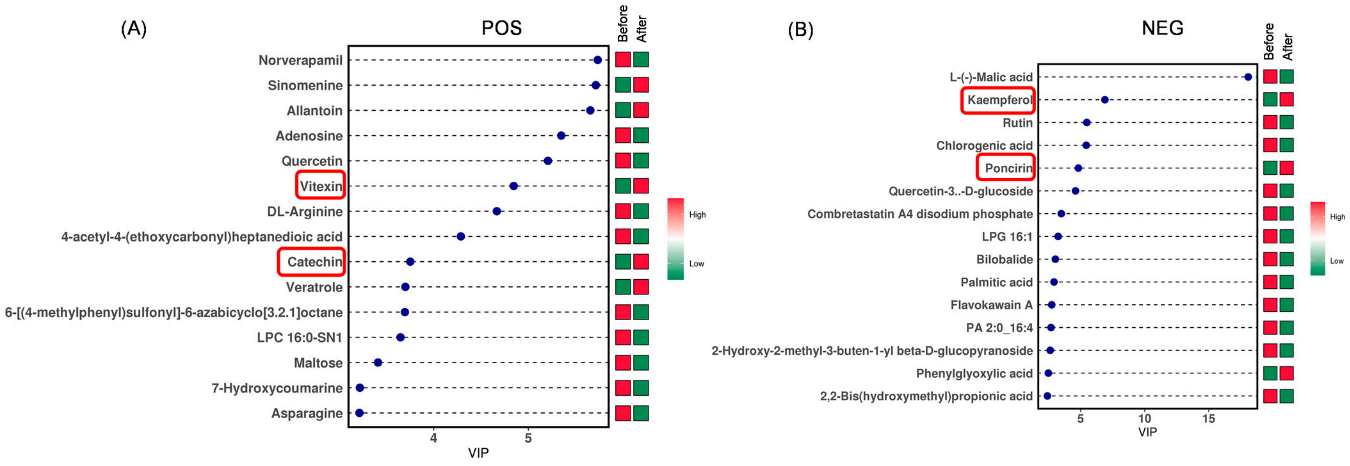

Figure S1. Differential metabolites before and after fermentation. (A) Positive ion mode; (B) Negative ion mode.

Institutional ethics guidelines

实验动物伦理审查证明

兹有 食品科学学院 项目负责人 阚建全 开展的 基于青花椒叶成分转化的功能微生物筛选及功能性富酶生物饲料产品研发 中的动物实验，在我校实验动物设施完成，项目全程受到西南大学实验动物伦理审查委员会的监督，并通过了伦理审查，伦理审查号为 IACUC-20240410-01

日期： 2024-10-29

西南大学实验动物伦理审查委员会(章)

Certificate of Laboratory Animal Ethics

IACUC No. Approved: IACUC-20240410-01

Institution: College of Food Science

Project: Screening of functional microorganisms based on the conversion of green peppercorn leaves and development of functional enzyme-enriched biofeed products , Animal Experiment Protocol.

Principle investigator: Jianquan Kan .

This Project has been supervised and approved by Institutional Animal Care and Use Committee of Southwest University.

Date: 2024-10-29

Institutional Animal Care and Use Committee (IACUC) of Southwest University
